# Hybridization generates a hopeful monster: a hermaphroditic selfing cichlid

**DOI:** 10.1098/rsos.150684

**Published:** 2016-03-23

**Authors:** Ola Svensson, Alan Smith, Javier García-Alonso, Cock van Oosterhout

**Affiliations:** 1 School of Biological, Biomedical and Environmental Sciences, University of Hull, Hull, UK; 2 Department of Biological and Environmental Sciences, University of Gothenburg, Box 463, Gothenburg 405 30, Sweden; 3 Biodiversity Group, Centro Universitario Regional Este, Universidad de la República, Maldonado 20000, Uruguay; 4 School of Environmental Sciences, University of East Anglia, Norwich Research Park, Norwich, UK

**Keywords:** colour polymorphism, disorders of sex development, *Pundamilia pundamilia*×*Neochromis omnicaeruleus*, self-fertilization, selfing, transgressive segregation

## Abstract

Compared with other phylogenetic groups, self-fertilization (selfing) is exceedingly rare in vertebrates and is known to occur only in one small clade of fishes. Here we report observing one F_1_-hybrid individual that developed into a functional hermaphrodite after crossing two closely-related sexually reproducing species of cichlids. Microsatellite alleles segregated consistent with selfing and Mendelian inheritance and we could rule out different modes of parthenogenesis including automixis. We discuss why selfing is not more commonly observed in vertebrates in nature, and the role of hybridization in the evolution of novel traits.

## 1. Introduction

Functional hermaphroditism is common in both plants and animals but is exceedingly rare among vertebrates [1]. The vertebrate exception is teleost fishes in which functional hermaphroditism has been reported in 27 families in seven orders, predominantly among tropical marine perciforms [2]. Self-fertilization (selfing) is much rarer than hermaphroditism, although this too is found in many animal and plant taxa [3]. In vertebrates, there are more than 80 known taxa of vertebrates that reproduce without sex [4], whereas selfing has only been observed in the clade with two species of *Kryptolebias* [2,5]. In teleost fish, all known parthenogenic lineages have hybrid origin [6].

In cichlids, the heterogametic sex differs between species, and the sexes are environmentally determined in some species (e.g. by pH or temperature), which can occur even in the presence of genetic sex determination [7]. Sex-determination systems turnover in conjunction with female orange blotch (OB) polymorphism are thought to have contributed to the formation of haplochromine cichlid species flocks in the African great lakes [8,9]. Sex change has been suggested in *Crenicara punctulata* [10], and in *Cichlasoma portalegrense* there is a description of a fully developed intersex gonad [11]. Here we report an individual cichlid that was produced in an F_1_-hybrid cross in a study on the OB polymorphism and sex determination. Uniquely, this individual reproduced fertile female and male offspring by hermaphroditic selfing.

## 2. Material and methods

**Figure 1.**
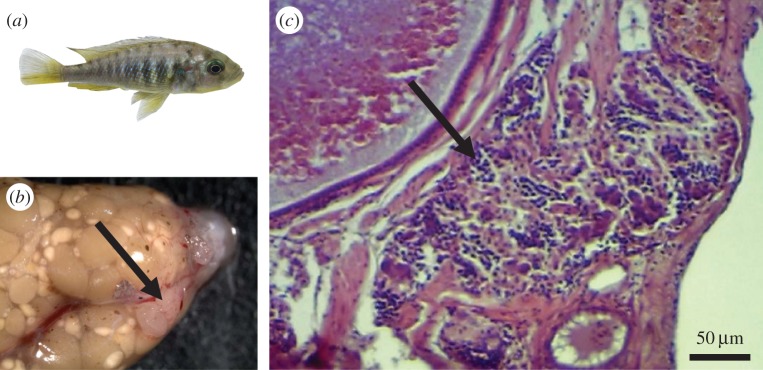
(*a*) The intersex produced by a cross between a female *Pundamilia pundamilia* from Python Island and an orange blotch (OB) *Neochromis omnicaeruleus* male from Makobe Island. The gonads (*b*) looked like typical cichlid ovaries. However, a horizontal plane section (*c*) contained small aggregated dark dots that resembled spermatocytes (marked by arrow). On the gonad (*b*), this section was located in a whitish tissue (arrow).

Females of *Pundamilia (Haplochromis) pundamilia* and *Pundamilia (Haplochromis) nyererei* were crossed with OB-coloured males of *Neochromis (Haplochromis) omnicaeruleus* (*N* = 31 crosses). These closely-related, often sympatric, species have an estimated divergence time of 0.1–0.35 million years [12]. When 81 F_1_-female hybrids were kept in isolation for one to six weeks (to be photographed), one female (‘intersex’, *P. Pundamilia* × *N. omnicarelueus*, figure 1*a*) spawned. In total, this intersex produced 14 broods, raising 46 living F_2_-juveniles over a period of 25 months while in isolation in a 20 × 20 × 20 aquarium. Two sons and 15 daughters survived to adulthood and were fertile. The intersex was then euthanized using an overdose of MS222 for morphological and histological analyses. As an effort to find further selfing individuals, all 18 full-sib sisters and 12 daughters of the intersex were kept in isolation for 12 months.

The intersex, its parents and the first four offspring were genotyped at nine microsatellite loci (Ppun5, Ppun7, Ppun17, Ppun21, Ppun32, Pzeb1, UME003, UNH002 and UNH130). Methods for DNA extraction and PCR reactions were as described previously [13,14]. Samples were genotyped on a Beckman Coulter CEQ 8000 capillary sequencer and genotypes were determined manually. We wrote a computer model to investigate whether the observed heterozygosity (Ho) of the offspring was consistent with selfing, or whether inbreeding with a (simulated) full sibling could also explain Ho. The model calculates Ho across nine microsatellites of four simulated offspring and assumes that the intersex produced with a simulated full sib (generated by the model based on the parents' genotypes). This procedure was repeated 10 000 times. The Ho of the four genotyped offspring was then compared with the distribution of simulated Ho values.

The gonads were fixed for 24 h in Bouin's solution or 4% paraformaldehyde solution, embedded in paraffin and serially sectioned (horizontal plane) at 5 and 7 µm. Sections were stained with haematoxylin and eosin to identify oogenic and spermatogenic tissue. Slides were examined with an Axiolab (Zeiss) light microscope and photographed.

## 3. Results

All alleles of each offspring were present in the intersex (table 1) and together with the fact that the intersex was held in isolation, we can for each analysed offspring confidently exclude all known modes of reproduction in vertebrates except selfing. Other forms of reproduction without a mate would result in either genetically identical clones, or individuals with a near-complete homozygous genome (automixis with terminal meiotic fusion) or heterozygous genome (automixis with central fusion) [6,15–19]. The intersex was heterozygous for the nine microsatellite loci and the mean observed heterozygosity (Ho) of the four offspring at these loci was Ho = 0.444 (table 1). This value is approximately equivalent to the level of heterozygosity expected from selfing of a completely heterozygous individual (Ho = 0.5). We modelled the distribution of Ho values based on the simulated full-sib mating in a computer model which showed that the observed value is significantly lower than the simulated heterozygosity (Ho (5–95% CI) = 0.746 (0.611–0.861); *p* < 0.001). Thus, the observed level of inbreeding was significantly higher than inbreeding resulting from non-selfing sexual reproduction, even if mating had occurred with the most closely-related individual possible (i.e. a full sibling).

**Table 1.** Microsatellite DNA analysis.

individual	Pmin5	Pmin7	Pmin17	Pmin21	Pmin32	Pzeb1	UME003	Unh002	UNH130
mother	204/220	217/261	105/113	356/377	155	155/183	245/262	228	215/219
father	196/236	241/269	117/133	317/365	157/161	231	241/249	220	181/188
intersex	220/236	217/269	105/133	317/377	155/157	183/231	245/249	220/228	188/219
offspring 1	236	217	105	377	155/157	183/231	245/249	220/228	188
offspring 2	236	269	105/133	377	155/157	183/231	249	220	219
offspring 3	236	217/269	133	317/377	155	183/231	249	220/228	219
offspring 4	220/236	217/269	133	317	155/157	231	245/249	220/228	219

The ovaries of the intersex female were located dorsally to the gut and filled a large volume of the peritoneal cavity. In the histological samples, ovarian tissue contained oogonia and oocytes in all stages of germ cell development. However, a whitish tissue in the antero-ventral part of the gonad (figure 1*b*) without any apparent barrier to the ovarian tissue showed an unusual aggregation of small dark dots. These dots had an appearance similar to spermatocytes (figure 1*c*).

### 3.1. Adult offspring

Of the 46 F_2_-offspring of the intersex female, three developed male nuptial coloration, and 15 showed female nuptial coloration after 12 months, which deviates significantly from an equal sex ratio (binomial test, *p* = 0.0075). Twenty-eight individuals could not be sexed because they died when juveniles (58.5% mortality), which was higher than the (mean ± s.d.) 7.6 ± 13.1% mortality in F_1_-crosses between the same two species as the intersex female (RB Stelkens, personal communication, [20]). When crossed, both surviving F_2_-males successfully spawned with 10 of the 15 sisters and produced offspring of both sexes with an approximately equal sex ratio (15 males and 18 females, binomial test, *p* = 0.73). This F_3_-generation also showed reduced survival (45% juvenile mortality), but proved to be fertile. None of the 18 full-sib sisters of the intersex, nor any of the 12 daughters, reproduced whilst kept in isolation.

## 4. Discussion

An individually housed F_1_-hybrid cichlid (intersex) produced viable and fertile offspring of both sexes without access to sperm from another individual. Microsatellite DNA analyses showed no evidence for aberrant chromosome numbers, i.e. the intersex and its offspring were diploid, and their observed heterozygosity (Ho) was consistent with Mendelian segregation after self-fertilization (selfing). Furthermore, the Ho was significantly lower than a hypothetical single generation of full-sib mating (i.e. the observed level of inbreeding was more severe). Sexually reproducing vertebrates occasionally produce offspring through facultative parthenogenesis [18,19], which appears to be realized through automixis where the terminal meiotic products fuse causing genome wide homozygosity [18,19]. Central fusion automixis is not known in vertebrates and typically all offspring are females with extensive genome wide heterozygosity [15–17]. Hence, both modes of automixis can be rejected. The gonads of the intersex resembled those of normal cichlid ovaries [21], and although the histology was inconclusive, it revealed a tissue containing clusters of dark dots which resembled spermatocytes. Given these lines of evidence, we conclude that the multiple batches of progeny of the intersex were produced by hermaphroditic selfing.

Only one previous study reported an intersex individual guppy (*Poecilia reticulata*) that may have reproduced by selfing [22]. Given that intersex individuals are often discovered by dissection, and because it is unusual to keep individuals in isolation, it is likely that the incidence of hermaphroditic selfing is currently underestimated. Intersex in hybrids have been reported previously; bitterling hybrids of the genus *Acheilognathus*, *Rhodeuscan* and *Tanakia* get distorted sex ratio and intersex [23], which was thought to be caused by abnormal interaction between sex-determination genes. Because OB-polymorphism appears to be involved in the reversal of genetic sex determination in cichlids [8,9], the parents of the intersex female (non-OB × OB) were likely to possess different sex determining genes. However, selfing appears to be an oddity rather than a rule in this cross because none of the intersex’ sisters or offspring reproduced by selfing when kept in isolation. Nevertheless, rare hybridization-fuelled ‘innovation’ could have adaptive potential by generating ‘hopeful monsters' with novel traits for selection to act upon [12,24–26]. Even though the long-term evolutionary potential of these ‘hopeful monsters’ is doubtful, there will be conditions in nature that may increase the likelihood for them to be biologically significant, e.g. when the environmental conditions change. During range expansion and colonization of novel habitats, hybridization is more frequent [27] and rare selfing immigrants may establish novel evolutionary lineages when offspring revert to (gonochoristic)\break sexual reproduction.

Why is selfing not more commonly observed in vertebrates in nature? Developmental or morphological constraints involved in ovotestis development [28] or hormonal constraints in simultaneously developing eggs and sperm [29] may explain the absence of hermaphroditism and selfing in mammals and birds. In teleosts, however, hermaphroditism is relatively common and intersex does occasionally occur in nature [2,30], which suggests that selfing lineages do evolve. Yet, a selfing lineage is likely to be short-lived because recessive deleterious mutations will become expressed in homozygous state which increases the extinction risk [31]. Given that many vertebrate species have a low reproductive potential, selection will be inefficient in purging deleterious mutations from a selfing lineage. Indeed, as in many selfing plants [31], rather than obligate selfing, the only known selfing vertebrates (*Kryptolebias marmoratus* and *K. hermaphroditus*) have evolved a mixed mating strategy of predominant selfing with occasional outcrossing [32], which may partially offset the deleterious consequences of selfing. In the present study, the offspring of the intersex female appeared to suffer from inbreeding depression resulting in high juvenile mortality. Furthermore, there was a significant female-biased sex ratio in those F_2_-hybrids. Such bias in sex ratio is consistent with Haldane's rule [33] which stipulates that the heterogametic sex tends to be absent or rare in hybrids. However, because the parents of the intersex female (non-OB × OB) were likely to possess different sex determining genes with different heterogametic sexes [8,9], and because the F_3_-hybrids showed no deviation in sex ratio, we do not want to speculate whether or not the observed female bias in the F_2_-hybrids is due to Haldane's rule, aggression based sex biased mortality, or deviation due to conflicting sex-determination systems.

To conclude, one F_1_-hybrid individual developed into a functional selfing hermaphrodite after crossing two closely-related sexually reproducing species of cichlids. Hermaphroditic selfing is likely to be underreported in vertebrates because of the unusual set of conditions that is required for it to be observed. Our study shows that facultative selfing is possible in teleost fishes, and we suggest that although such ‘hopeful monsters’ may be evolutionarily transient and rare in nature, certain environmental conditions may make them biologically significant.

## Data accessibility

All raw data are included in the article. The model code is available from C.v.O. upon request.
